# Performance Comparison of the *artus* HBV QS-RGQ and the CAP/CTM HBV v2.0 Assays regarding Hepatitis B Virus DNA Quantification

**DOI:** 10.1155/2020/4159189

**Published:** 2020-04-14

**Authors:** Salma Madihi, Hashim Syed, Fatiha Lazar, Abdelmajid Zyad, Abdelouaheb Benani

**Affiliations:** ^1^Molecular Biology Laboratory, Pasteur Institute of Morocco, 1, Place Louis Pasteur, Casablanca 20360, Morocco; ^2^Team of Experimental Oncology and Natural Substances, Cellular and Molecular Immunopharmacology, Sultan Moulay Slimane University, Faculty of Sciences and Technologies, Beni Mellal, Morocco

## Abstract

**Background:**

Over 240 million people are chronically infected with hepatitis B virus (HBV), the leading cause of liver cancer worldwide. The quantification of the HBV DNA level is critical for monitoring the efficacy of antiviral treatment of chronic HBV patients.

**Methods:**

In our study, we compared the performance of the *artus* HBV QS-RGQ assay to the CAP/CTM v2.0 test, as a reference method, on 142 Moroccan patients. The analytical performance of the *artus* HBV QS-RGQ assay, such as the limit of detection, quantification, precision, reproducibility, and linearity, was determined using dilution series from 10 to 0.1 log_10_ IU/mL.

**Results:**

Detection rates and viral loads quantified by the *artus* HBV QS-RGQ assay were significantly lower than those from the CAP/CTM v2.0 assay (73.94% vs. 82.39%; 3.34 ± 1.94 log_10_ IU/mL vs. 3.91 ± 2.45 log_10_ IU/mL; *p* < 0.01). A Bland-Altman plot found a mean difference of (CAP/CTM v2.0 − *artus* HBV QS − RGQ) = 0.5717 log_10_ IU/mL, with an average range of -1.13 to 2.31 log_10_ IU/mL. The two methods demonstrated a high correlation (*r* = 0.88) for 100 positive samples, a moderate correlation for samples below 2000 IU/mL (*r* = 0.76), and a very high correlation for the samples above 2000 IU/mL (*r* = 0.95). Linearity of the *artus* QS-RGQ test ranged from 1.07 to 7.51 log_10_ IU/mL.

**Conclusion:**

The *artus* HBV QS-RGQ assay showed a strong correlation, precision, and linearity in comparison with the CAP/CTM v2.0. However, viral loads determined by the *artus* HBV QS-RGQ assay were lower than those determined by the CAP/CTM v2.0 assay.

## 1. Introduction

Viral hepatitis B is a prevalent human infection and a global health problem. Worldwide, about 240 million people are HBV surface antigen- (HBsAg-) positive chronic carriers [1]. The prevalence in Morocco is estimated to about 1.81% [2]. HBV is highly infectious and may be transmitted via blood or sexual contact, leading to chronic infection, progressive liver damage, hepatocellular cancer (HCC), and death. Chronic infection is most likely to occur due to perinatal infection from infected mothers or in early childhood via horizontal transmission, when the immune system is not yet mature [3]. The WHO reported that complications related to chronic HBV infection are a major source of morbidity and mortality and estimated the annual number of HBV-related deaths from liver cirrhosis and HCC to be 1.34 million deaths per year [4]. In the past, HBeAg and anti-HBe, as serological markers, had allowed detecting infectivity and viral replication, but their use for this purpose has been replaced by HBV DNA quantitation, which has changed the concept of nonreplicative infection and became an alternative and a reliable marker of replication activity. HBV DNA quantification is also an important tool for monitoring disease progression and assessing the response to antiviral therapy. Additionally, it had been reported that higher titers of HBV DNA are directly related to a rapid progression of the disease and high incidence of HCC [5]. Thus, early and powerful diagnosis is needed. In fact, quantitative real-time PCR (qPCR) is currently a strong molecular tool and well-established method for quantification, detection, and typing of different pathogens, especially viral ones. It plays a critical role in the management of chronic HBV infections thanks to its increased accuracy, wider linear range, and reproducibility [6]. Several commercial assays for HBV DNA quantitation have been described; thus, medical laboratories often need to evaluate the agreement between two measurement methods in order to choose the best one. By this study, we aimed to assess the performance of the *artus* HBV QS-RGQ (QIAGEN) assay in comparison with the COBAS AmpliPrep/COBAS TaqMan HBV v2.0 abbreviated to CAP/CTM HBV v2.0 (Roche Molecular Diagnostics) assay, on clinical Moroccan samples.

## 2. Methods

### 2.1. Samples

A total of 142 HBsAg-positive samples, using an automatic chemiluminescence immunoassay analyzer (Architect, Abbott Laboratories, USA), were included in the current study. All samples were collected between May 2018 and May 2019 at the molecular biology laboratory at the Institute Pasteur of Morocco (Casablanca, Morocco). Samples were quantified by the Roche CAP/CTM v2.0 platform, as a routine practice. Analyzed plasma was separated from EDTA whole blood, and the remaining amounts were stored at -20°C and analyzed by the QIAGEN EZ1 Advanced XL/Rotor-Gene Q platform. Serologically testing, all patients had been diagnosed positive for HBsAg.

### 2.2. qPCR Assays

#### 2.2.1. The Roche COBAS AmpliPrep/COBAS TaqMan HBV Test v2.0

The CAP/CTM HBV v2.0 assay (Roche Molecular Diagnostics) is a fully automated viral load quantitative hepatitis B test used in the management of patients with chronic hepatitis B infection undergoing antiviral therapy. The test treats 650 *μ*L of the serum or plasma sample. In our case, we used 650 *μ*L of plasma samples and the nucleic acid was extracted in 45 min using magnetic particle technology. HBV DNA (50 *μ*L) was analyzed by the COBAS TaqMan 48, system for real-time automated amplification, and nucleic acid detection using primers and probes targeting the highly conserved precore and core region. Amplification concerns two targets: HBV DNA and the internal quantitation standard (QS), and results were given in IU/mL with a conversion factor of 5.82 copies/IU of HBV DNA. The CAP/CTM HBV v2.0 system offers a broader dynamic range, from as low as 2 × 10^1^ IU/mL to as high as 1.7 × 10^8^ IU/mL (1.3–8.2 log_10_ IU/mL), according to the manufacturer and required 150 min to be amplified and detected by the COBAS TaqMan 48.

#### 2.2.2. The *artus* HBV QS-RGQ Assay

HBV DNA was extracted in 43 min from 400 *μ*L of plasma samples using the EZ1 advanced XL instrument, using the EZ1 DSP virus kit (QIAGEN) based on magnetic bead technology. For each HBV plasma sample, a premix of 7.9 *μ*L internal control, 4 *μ*L of cRNA, and 54.1 *μ*L of AVE were added to the elution tube, resulting in a final volume elution of 60 *μ*L. 20 *μ*L of extracted HBV DNA were added to 30 *μ*L of the master mix, containing all necessary reagents and enzymes for specific amplification of a 134 bp region of the HBV core gene, provided by the *artus* HBV QS RGQ kit. PCR reaction was insured by the Rotor-Gene Q platform for 107 min. Results were provided in IU/mL with a conversion factor of 8.21 copies/IU. The linear range offered by the *artus* HBV QS-RGQ assay covers concentrations from 3.16 × 10^1^ IU/mL to 2 × 10^7^ IU/mL (1.5–7.3 log_10_ IU/mL), and analytical sensitivity (LOD) is 10.21 IU/mL, according to the manufacturer.

### 2.3. Criteria for Interpreting the Viral Load of HBV DNA

Interpretation of the HBV viral load results is based on the limit of detection (LOD) of each assay and was recorded either as target detected or as target not detected, while the quantitative result concern was conducted only to patients with viral loads above 2 × 10^1^ IU/mL for CAP/CTM HBV v2.0 and 3.16 × 10^1^ IU/mL for *artus* HBV QS-RGQ, in the current study.

### 2.4. Statistical Analysis

All results were converted to IU/mL and transformed to log_10_ for further statistical analysis using IMB SPSS Statistics 25 (International Business Machines Corp., Armonk, NY, USA). The Spearman correlation coefficient (*r*) was calculated to determine the linear relationship between the two assays, and the Bland-Altman analysis was used to assess the agreement between the two methods of viral load measurement [7]. The bias between the assays was calculated as the mean *m* of the difference between the two measurements (CAP/CTM HBV v2.0-artus HBV QS-RGQ), and its standard deviation (SD) was calculated. The 95% limits of agreement between the assays were determined as *m* ± 1.96 SD. The SD for the two methods (CAP/CTM HBV v2.0, artus HBV QS-RGQ) was plotted against the average of the two measurements ((CAP/CTM HBV v2.0-artus HBV QS-RGQ)/2). For each sample, the largest difference in HBV DNA levels between the two methods was classified according to three classes: ≤0.5, [0.5-1], and >1 log_10_ IU/mL. For all analyses, *p* values < 0.05 with the two-tailed test referred to statistical significance.

## 3. Results

### 3.1. Agreement and Correlation between the *artus* HBV QS-RGQ Assay and the CAP/CTM HBV v2.0

A total of 142 samples were used in this study to evaluate the correlation between the *artus* HBV QS-RGQ and the CAP/CTM HBV v2.0 assays. Twenty samples yielded undetectable loads on both assays. Thirty-seven samples showed undetectable results (<31.6 IU/mL) using the *artus* HBV QS-RGQ assay, 17 of which generated quantitative results on the CAP/CTM HBV v2.0 (≥20 IU/mL). However, one sample, undetectable on the CAP/CTM HBV v2.0, was measured using the *artus* HBV QS-RGQ assay and generated a low signal. This result was verified, by double reextraction of the sample followed by its amplification five times. The obtained signals for such sample were between 6.06 IU/mL and 12.7 IU/mL (Table 1).

Concerning high viral loads, two samples showed concentrations above the maximal value of detection (1.7 × 10^8^ IU/mL) using the CAP/CTM v2.0. To determine the actual sample concentration, the two samples were diluted 10- and 100-folds and the concentrations were calculated as 1.28 × 10^10^ IU/mL (10.11 log_10_ IU/mL) and 1.28 × 10^9^ IU/mL (9.11 log_10_ IU/mL), respectively. The two samples were tested 3 and 4 times using the *artus* HBV QS-RGQ assay, respectively. For each sample, two of the runs resulted in no given concentrations. The four missing values were determined using the five HBV quantitation standard graphs (concentration and Ct) which range from 10^4^ IU/mL to 10^8^ IU/mL. The results were 3.01 × 10^7^ IU/mL (7.47 log_10_ IU/mL), 2.57 × 10^7^ IU/mL (7.41 log_10_ IU/mL), 3.18 × 10^7^ IU/mL (7.5 log_10_ IU/mL), and 3.22 × 10^7^ IU/mL (7.51 log_10_ IU/mL), respectively.

**Figure 1:**
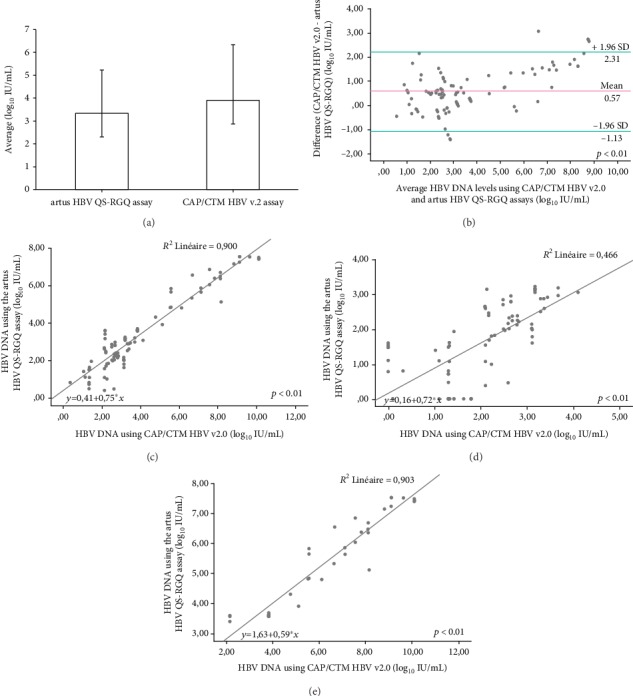
Comparison of HBV DNA levels between the CAP/CTM HBV v2.0 and the *artus* HBV QS-RGQ assays. (a) Comparison of means and deviation of the *artus* HBV QS-RGQ and the CAP/CTM HBV v2.0 assays, (b) Bland Altman plot of CAP/CTM v2.0-artus HBV QS-RGQ (*n* = 100; *p* = 0.00), (c) Passing–Bablok regression of positive samples (*n* = 100; *p* = 0.00), (d) Passing–Bablok regression of viral loads below 2000 IU/mL (*n* = 84; *p* = 0.00), and (e) Passing–Bablok regression of viral loads above 2000 IU/mL (*n* = 38; *p* = 0.00).

Among the 142 tested samples, HBV DNA was quantified in 105 samples (73.94%) by the *artus* HBV QS-RGQ assay and 117 samples (82.39%) by the CAP/CTM HBV v2.0 assay. The mean ± SD of the HBV DNA level of detection was 3.34 ± 1.94 log_10_ IU/mL for the *artus* HBV QS-RGQ assay and 3.91 ± 2.45 log_10_ IU/mL for the CAP/CTM HBV v2.0 assay (Figure 1(a)). The limits of agreement, determined as ±1.96 SD, were from -0.46 to 7.14 and from -0.89 to 8.71 for the *artus* HBV QS-RGQ and the CAP/CTM HBV v2.0 assays, respectively. Overall, the viral loads quantified by the *artus* HBV QS-RGQ assay were significantly lower than those quantified by the CAP/CTM HBV v2.0 assay (*p* < 0.01).

One hundred samples yielded positive results for both assays (≥20 IU/mL for the CAP/CTM HBV v2.0 and ≥31.6 IU/mL for the *artus* HBV QS-RGQ assay) and were uniformly distributed between 20 IU/mL and 1.28 × 10^10^ IU/mL. The Bland-Altman analysis revealed the mean difference between the two assays (CAP/CTM HBV v2.0–*artus* HBV QS-RGQ) as *m* = 0.5717 log_10_ IU/mL, with an average range ± 1.96 SD of -1.13 to 2.31 log_10_ IU/mL (SD = 0.87) (*p* < 0.01) (Figure 1(b)). The Spearman's coefficient (*r* = 0.88) showed a strong correlation between the CAP/CTM HBV v2.0 and the *artus* HBV QS-RGQ assays ([*artus* HBV QS − RGQ] = 0.4051 + 0.75 [CAP/CTM HBV v2.0]; 95% confidence interval (CI) of intercept: 0.179 to 0.636; slope: 0.7 to 0.8; *R*^2^ = 0.90) (*p* < 0.01) (Figure 1(c)). Among the positive samples, 47% (*n* = 47) were ≤0.5 log_10_ IU/mL of difference between the two methods, 20% (*n* = 20) were between 0.5 and 1 log_10_ IU/mL, and 33% (*n* = 33) were >1 log_10_ IU/mL. Six samples were outliers (6%), three of which read higher on the CAP/CTM HBV v2.0.

Eighty-four samples were found positive in at least one assay, with HBV DNA levels < 2000 IU/mL. They were quantified by both assays and showed moderate correlation (Spearman's coefficient: *r* = 0.76; *p* < 0.01) and a linear regression's equation as follows: [*artus* HBV QS − RGQ] = 0.157 + 0.721 [CAP/CTM HBV v2.0]; 95% CI of intercept: -0.234 to 0.548; slope: 0.551 to 0.891; *R*^2^ = 0.47 (Figure 1(d)). The Bland-Altman plot analysis of HBV DNA levels below 2000 IU/mL was elaborated resulting in a mean difference between the two assays of 0.4196 log_10_ IU/mL with an average range ± 1.96 SD of -1.22 to 2.06 log_10_ IU/mL (*p* < 0.01). Conversely, samples with HBV DNA levels above 2000 IU/mL (*n* = 38) showed a high correlation between the two assays (Spearman's coefficient: *r* = 0.95; linear regression equation: [*artus* HBV QS‐RGQ] = 1.626 + 0.594 [CAP/CTM HBV v2.0]; 95% CI of intercept: 1.185 to 2.067; slope: 0.528 to 0.660; *R*^2^ = 0.90) (Figure 1(e)). The Bland-Altman plot analysis of HBV DNA levels above 2000 IU/mL was elaborated resulting in a mean difference between the two assays of 0.91 log_10_ IU/mL with an average range ± 1.96 SD of -1.25 to 3.1 log_10_ IU/mL (*p* < 0.01).

### 3.2. Performance of *artus* HBV QS-RGQ versus CAP/CTM HBV v2.0

**Figure 2:**
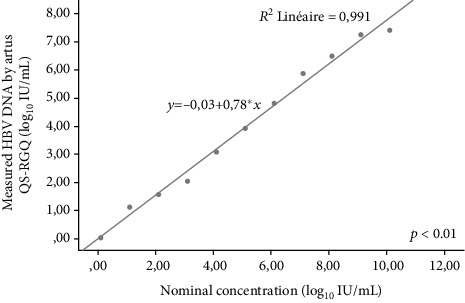
A linear regression analysis of the HBV DNA load determination using the *artus* HBV QS-RGQ assay (*p* = 0.00).

The linearity using a ten-member panel was generated from a serial dilution of high-titer specimens from 10 to 0.1 log_10_ IU/mL in duplicate per each concentration. Each dilution was prepared using 0.1 mL of the sample and 0.9 mL of HBV-negative plasma. Results ranged between 1.07 and 7.51 log_10_ IU/mL. The straight line and regression statistics were determined using a linear regression of the log_10_-calculated concentrations with the log_10_ nominal concentrations: *y* = −0.03460 + 0.7779 *x*; *R*^2^ = 0.9908 (Figure 2).

To evaluate the precision and reproducibility of the *artus* HBV QS-RGQ assay in comparison with the CAP/CTM HBV v2.0 assay, eight samples were measured more than three times over three days by both assays, with different HBV DNA levels described as high (≥1 × 10^7^ IU/mL), medium (1 × 10^7^ to 2 × 10^3^ IU/mL), and low (<2 × 10^3^ IU/mL) HBV DNA titers. The SD and the coefficient of variation (CV) were determined. The CV ranged from 0.62% to 81.23% and from 0.00% to 3.35% for the *artus* HBV QS-RGQ and the CAP/CTM HBV v2.0 assays, respectively (Table 2).

## 4. Discussion

Despite the access to effective antiviral drugs and vaccines, HBV infection remains a major public health challenge worldwide. HBV DNA loads are detectable in the blood at the early stage of infection (1 month after HBV infection) and, during the chronic infection, can vary from undetectable to more than 10^10^ IU/mL. To predict the stage of HBV infection or progression of liver disease in HBV-infected individuals, HBV DNA load quantitation is needed and serves as a strong and crucial tool for the initiation of treatment or for the therapeutic follow-up, as low residual amount of HBV DNA may persist after treatment leading to relapse, recurrence, and drug resistance [8]. To the end of this point, several commercially qPCR assays have been developed. For the best of our knowledge, this study is the first in Morocco and Africa to compare results of HBV DNA viral loads using the two methods *artus* HBV QS-RGQ and the CAP/CTM v2.0, which is the predominantly used method in the routine diagnosis of HBV.

In our study, a strong overall correlation and agreement between the two assay methods (*r*^2^ = 0.90) was detected. Our results were similar to those reported by Brichler et al. who found a good correlation and agreement of *r*^2^ = 0.89 between the two methods [9]. In 2017, a study was performed by Han et al. also showed a high correlation, *r*^2^ = 0.86, and agreement between the two assays [10].

Overall, 47% of samples showed ≤0.5 log_10_ IU/mL of difference between the two methods. This difference is not clinically significant and does not affect the results. In this regard, Pawlotsky reported that differences or variations of less than 0.5 log_10_ IU/mL should not be taken into account, as they may be due to intrinsic or between-patient variability [11]. However, a statistically significant difference in the quantification of HBV DNA levels was observed in our clinical samples, where 20% were between 0.5 and 1 log_10_ IU/mL of difference, revealing higher HBV DNA loads quantified by the CAP/CTM HBV v2.0 in comparison with the *artus* HBV QS-RGQ. Those results are close to the results reported by Brichler et al., who found that the difference in 23% of the samples exceeded ±0.5 log_10_ IU/mL and concluded that HBV DNA loads by the *artus* HBV QS-RGQ assay were lower than the results by the CAP/CTM HBV v2.0 assay [9]. In addition, Yeh et al., in their comparison between the RealTime assay and TaqMan assay, reported that 27.3% of samples had HBV DNA levels measured greater than 0.5 log_10_ IU/mL. In contrast, the difference in 33% of the samples in the current study was above 1 log_10_ IU/mL which disagree with Yeh et al. who found such a difference was recorded in only 8.6% of the examined samples [12]. Additionally, 11.97% (*n* = 17) yielded detectable results only with the CAP/CTM HBV v2.0, while 3.5% of samples (*n* = 5) yielded detectable low signals only with the *artus* HBV QS-RGQ assay. The *artus* HBV QS-RGQ manufactures report that, while testing their HBV DNA load samples in comparison with the CAP/CTM HBV v2.0, 3 of 189 samples (1.59%) were detected only by the *artus* HBV QS-RGQ. This difference in HBV DNA load detection might be explained by the low HBV load or mutations in the precore and core promoter regions, which are highly prevalent in Morocco [13, 14]. Moreover, it was reported that the CAP/CTM HBV v2.0 test provided a genotype inclusivity for accurate viral load monitoring in serum and EDTA plasma samples (20 IU/mL for genotypes C, D, F, and G and the precore mutant versus 15 IU/mL for genotypes A, B, E, and H) [15]. Other studies showed that genotype B needs further investigation in the association with discordant results > 1 log_10_ IU/mL [12]. Although the genotype of the HBV-positive samples was not determined in the current study, however, studies have shown that more than 90% of HBV-infected people in Morocco belong to genotype D [16, 13, 17, 14].

Hepatitis B infection has a broad virological and clinical spectrum while antiviral therapy is not indicated for all patients. According to EASL 2017 recommendations, patients who have HBV DNA > 2000 IU/ml and, at least, moderate fibrosis may initiate treatment even if alanine aminotransferase (ALT) levels are normal, whereas patients with HBeAg-negative chronic HBV infection and HBV DNA ≥ 2000 IU/mL should be followed with ALT determinations at least every 3 months for the first year and every 6 months for at least 3 years [1]. In our study, we divided the patients into two categories: patients with HBV DNA below 2000 IU/mL (*n* = 84) and above 2000 IU/mL (*n* = 38). As results, we found that the HBV DNA levels determined by the *artus* HBV QS-RGQ assay were lower than those determined by the CAP/CTM HBV v2.0 test, showing a moderate correlation for the low viral loads (<2000 IU/mL) estimated to *r* = 0.76, higher than the correlation found by Han et al. (*r* = 0.49), according to Spearman's coefficient [10]. Another study performed by Shin et al. showed a moderate correlation estimated to *r* = 0.71 between the COBAS 4800 HBV test and the CAP/CTM HBV v2.0 assay, comparing samples with HBV DNA levels below 2000 IU/mL [18]. Conversely, our study reported a strong correlation between the two assays in case of HBV DNA levels above 2000 IU/mL, estimated to *r*2 = 0.90. Medium to high viral loads mostly show a good correlation between two methods of measurements. Shin et al. also reported a strong correlation (*r* = 0.95) between the COBAS 4800 HBV test and the CAP/CTM HBV v2.0 assay for samples with HBV DNA levels above 2000 IU/mL [18].

Linearity of HBV DNA loads using the *artus* HBV QS-RGQ assay was about *r*^2^ = 0.991, close to that given by the manufacturer (*r*^2^ = 0.999).

The limitations of the current study included relatively low sample size and inability to genotype the samples.

## 5. Conclusion

In summary, results of viral loads quantified by the *artus* HBV QS-RGQ assay were lower than those quantified by the CAP/CTM HBV v2.0 test. However, this study demonstrated the satisfactory fastness and performance of the *artus* HBV QS-RGQ assay at quantifying viral HBV DNA and managing patients with chronic HBV infection. Other than that, the EZ1 DSP virus kit has the benefit of copurifying both viral RNA and DNA with one chemistry and one purification protocol.

## Figures and Tables

**Table 1:** Comparison of HBV DNA viral load results in 142 samples quantified by the CAP/CTM HBV v2.0 and the *artus* HBV QS-RGQ assays.

*artus* HBV QS-RGQ (IU/mL)	CAP/CTM HBV v2.0 (IU/mL)		Total
	<20	≥20	
<31.6 not detected	20	17	37
<31.6 detected	5	0	5
≥31.6	0	100	100
Total	25	117	142

**Table 2:** Precision and reproducibility of HBV DNA quantitation of the *artus* HBV QS-RGQ and the CAP/CTM HBV v2.0 assays.

Sample description (viral load)	Number of replicates	Precision					
		*artus* HBV QS-RGQ assay			CAP/CTM HBV v2.0 assay		
		Mean (log_10_ IU/mL)	SD	CV (%)	Mean (log_10_ IU/mL)	SD	CV (%)
High	3	7.42	0.05	0.62	10.11	0.02	0.20
	4	7.44	0.14	1.93	9.11	0.03	0.35

Medium	3	5.42	0.53	9.73	5.55	0.19	3.35
	9	3.59	0.04	1.09	3.81	0.03	0.72

Low	6	3.14	0.06	1.82	3.17	0.03	1.08
	4	2.84	0.10	3.36	2.65	0.04	1.43
	3	1.54	0.47	30.52	2.11	0.02	0.72
	6	0.45	0.36	81.23	1.3	0.00	0.00

## Data Availability

The data that support the findings of this study are available on request from the corresponding author [Salma Madihi]. The data are not publicly available due to them containing information that could compromise research participant privacy and/or consent.
